# Correlation of Serum Vaspin, Omentin-1, and adiponectin with metabolic phenotypes in Type-2 diabetes mellitus patients

**DOI:** 10.12669/pjms.37.7.4330

**Published:** 2021

**Authors:** Mukhtiar Baig, Zohair J Gazzaz, Marwan A Bakarman, Sami H Alzahrani

**Affiliations:** 1 Dr. Mukhtiar Baig, Ph.D. Department of Clinical Biochemistry, Faculty of Medicine, Rabigh, King Abdulaziz University, Jeddah, Saudi Arabia; 2 Dr. Zohair J Gazzaz, Ph.D. Department of Internal Medicine, Faculty of Medicine, Rabigh, King Abdulaziz University, Jeddah, Saudi Arabia; 3 Dr. Marwan A Bakarman, FFCM. Department of Family and Community Medicine, Faculty of Medicine, Rabigh, King Abdulaziz University, Jeddah, Saudi Arabia; 4 Dr. Sami H Alzahrani, SBFM Department of Family Medicine, Faculty of Medicine, King Abdulaziz University, Jeddah, Saudi Arabia.

**Keywords:** Diabetes Mellitus, Adipokines, Vaspin, Omentin-1, Adiponectin

## Abstract

**Objectives:**

To investigate adipocytokines’ (vaspin, omentin-1, and adiponectin) correlation with metabolic phenotypes in type 2 diabetes mellitus (T2DM) patients.

**Methods:**

This case-control research was done at the Diabetic Clinic in Jeddah, Kingdom of Saudi Arabia (KSA), from November 2018 to March 2019. Seventy-five T2DM patients and 75 gender, age, and BMI-matched healthy subjects were recruited for this research.

**Results:**

In DM patients, the concentrations of serum vaspin and omentin-1 were substantially lower (p<0.001) than in the control group. A significant positive relationship between vaspin concentration and DBP (p<0.001), BMI (p<0.001), and waist circumference (p<0.001) was found in patients and control subjects, while FPG (p<0.016), serum insulin (p<0.001), HOMA-IR (p<0.001), TC (p<0.001), TG (p<0.001), and LDLc (p<0.001) were significantly interrelated among patients. Serum concentrations of omentin-1 and ADN were significantly negatively correlated with serum insulin, HOMA-IR, and TG among the DM group. Serum vaspin and ADN levels were significantly higher in the cases and control groups with BMI>25, and no gender-wise variance was observed in adipocytokines levels. Binary logistic regression analysis showed a significantly negative predictive relationship of vaspin and omentin-1 with DM.

**Conclusion:**

The DM group displayed substantially lower serum vaspin and omentin-1 levels. However, there was no consistent relationship observed between these adipocytokines and metabolic phenotypes.

## INTRODUCTION

In the recent two decades, diabetes mellitus has doubled worldwide; therefore, it has become an essential challenge to public health. T2DM is a common endocrinological problem in KSA. A recent statement by the World Health Organization described that DM prevalence in the adult population is about 8.5% (422 million adults), while in KSA, its prevalence is about 14.4%.^1^ All these three factors have an influential function in the occurrence of metabolic syndrome (MetS) and further progression to T2DM. A Saudi study stated 25.4% and 25.5% of people were diabetes and had impaired FPG, respectively.^2^

Adiponectin (ADN) is an adipose-specific adipokine that induces the insulin-sensitizer effect. Among obese patients, ADN level is usually low, and giving it to obese subjects has reported an increase in insulin sensitivity.^3^ Adiponectin deficiency causes increased insulin resistance, while ADN over-expression leads to enhanced insulin sensitivity and glucose tolerance.^4^ Another adipocytokine known as Vaspin (visceral adipose tissue-derived serine protease inhibitor) has been potentially reported to have an insulin-sensitizer effect, and its gene expression is linked with obesity and MetS.^5^ Moreover, in the adipocyte-insulin axis, vaspin has been reported to play a part among T2DM patients having insulin resistance and those that are obese as well. Thus, vaspin can be associated with the metabolism of glucose and might substantially improve glucose tolerance and insulin sensitivity in patients.^6^ Levels of omentin-1 in plasma have been inversely correlated to BMI and waist circumference, fasting plasma insulin, and insulin sensitivity, ADN, and HDLc.^7^,^8^ Omentin-1 has been associated with enhancing insulin-mediated phosphorylation leading to the uptake of glucose and inhibiting TNF-α.^9^ This shows that omentin-1, like ADN, might be a protective adipokine.

In the literature, several adipokines have been implicated in the progression of DM. However, a disagreement in the literature is found regarding the involvement of serum omentin-1, vaspin, and ADN in T2DM and their relationship with a metabolic phenotype. So, we planned this study to find out their link with T2DM in a local population sample. The research objective was to identify the correlation of serum vaspin, omentin-1, and ADN with metabolic phenotype among T2DM patients.

## METHODS

This case-control research was performed at the Diabetic Clinic of a Primary Health Care Centre in Jeddah, KSA, from November 2018 to March 2019. Ethical approval was acquired from the Rabigh Faculty of Medicine-Research Ethics Review Committee Approval (No. RMC-01-37-H, dated 26 December, 2016) before the study’s start, and written consent was taken from all study participants. The blood sample and other data were collected from 75 adults (37 males and 38 females) with T2DM patients (age ranges from 40-60 years) and 75 healthy adult subjects (37 males and 38 females), comparable in gender, age, and BMI with patients. The control group was selected from the general population.

Subjects suffering from liver disease, chronic inflammatory disease, chronic kidney disease, or taking medications that could affect adipocytokines levels were excluded from the study. A proforma was filled regarding sociodemographic characteristics, and complete physical examination findings were recorded.

Height and weight were measured in meters and kilograms, respectively, and BMI was determined using the formula, and hip and waist circumference was also measured. HOMA-IR was approximated as “glucose (mmol/l* insulin (uU/ml), all divided by 22.5)”.^10^ For blood workup, a sample of five ml of venous blood in fasting was collected, and serum was stored for outcome parameters analysis. FPG, HbA1c, and lipid profile were determined in the King Abdulaziz University, Hospital on auto-analyzer. Human ELISA kits were utilized for measuring vaspin, omentin-1, ADN, and insulin levels.

SPSS version 26.0 was utilized for the data analysis. Quantitative data were reported as mean and standard deviation. Independent T-test was applied for detecting significance between the groups. Pearsons’ correlations were utilized to determine the correlation of adipocytokines with other variables. Logistic regression analysis was done to establish the association of study variables with DM. A relevant p-value of < 0.05 was counted significant.

## RESULTS

In DM patients, the concentrations of serum vaspin and omentin-1 were substantially lower (p<0.001), while no extensive variation in serum ADN levels was noted compared to the control group. The other baseline attributes of the participants are represented in Table-I.

**Table I:** General characteristics’ comparison of study groups.

*Parameters*	*Control group N=75*	*DM patients N=75*	*P-value*

	*Mean (SD)*	*Mean (SD)*	
Age (yrs)	54.15±6.06	53.16±8.74	0.871
BP Systolic (mm Hg)	125.92±10.13	139.25±17.41	0.000
BP Diastolic (mm Hg)	80.11±9.31	85.79±10.75	0.000
BMI (Kg/m^2^)	28.46±4.02	29.76±4.51	0.078
Waist circumf (in)	36.22±5.07	38.61±5.74	0.011
FPG (mmol/L)	5.07±0.26	8.12±1.57	0.000
Serum Insulin (mIU/ml)	7.98±2.83	17.43±4.52	0.000
HOMAIR	1.80±0.63	6.30±2.13	0.000
TC (mmol/l)	3.94±0.77	4.82±0.89	0.000
TG (mmol/l)	1.53±0.67	1.83±0.73	0.012
HDLc (mmol/l)	1.13±0.41	1.15±0.19	0.835
LDLc (mmol/l)	2.75±0.66	3.20±0.69	0.003
Vaspin (pg/ml)	336.92±58.51	260.30±75.64	0.000
Omentin-1 (ng/ml)	255.75±58.15	179.17±62.25	0.000
Adiponectin (ug/ml)	3.74±0.78	3.58±0.98	0.32

“BMI= Body mass index, FPG=Fasting plasma glucose, TC= Total cholesterol, TG=Triglycerides, LDLc= Lowdensity lipoprotein cholesterol, HDLc = High-density lipoprotein cholesterol”.

“BMI= Body mass index, FPG=Fasting plasma glucose, TC= Total cholesterol, TG=Triglycerides, LDLc= Low-density lipoprotein cholesterol, HDLc = High-density lipoprotein cholesterol”

A notable positive correlation was detected between levels of serum vaspin and DBP (p<0.001), BMI (p<0.001), and waist circumference (p<0.001) in patients and control subjects. At the same time FPG (p<0.016), serum insulin (p<0.001), HOMA-IR (p<0.001), TC (p<0.001), TG (p<0.001), and LDLc (p<0.001) were significantly correlated among patients only. Serum vaspin levels were inversely associated with omentin and positively linked with ADN among both groups (Table II). Serum omentin-1 had negative correlation with BMI among the control group and negatively related to waist circumference among both groups. A significant negative correlation of ADN and serum omentin-1 concentrations with serum insulin, HOMA-IR, and TG among the DM group was observed (Table II). Serum ADN was significantly correlated with DBP, BMI, and waist circumference in patients and the control group (Table-II).

**Table II:** Serum vaspin, omentin, and adiponectin levels relationship with biochemical and clinical parameters.

*Variables*	*Vaspin (pg/ml)*		*Omentin-1 (ng/ml)*		*Adiponectin (ng/ml)*	

	*Control (n=75)*	*Patients (n=75)*	*Control n=75)*	*Patients (n=75)*	*Control n=75)*	*Patients (n=75)*

	*r (P-value)*	*r (P-value)*	*r (P-value)*	*r (P-value)*	*r (P-value)*	*r (P-value)*
Age	-0.03(0.824)	-0.03(0.73)	0.03 (0.793)	0.03 (0.712)	-0.31(0.000)	-0.01 (0.69)
Systolic BP	0.44(0.001)	0.08(0.32)	-0.16(0.255)	0.15(0.124)	0.40 (.003)	0.09(.28)
Diastolic BP	0.44(0.001)	0.36(0.000)	-0.16(0.132)	-0.124(0.167)	0.41(.004)	0.33 (.000)
BMI	0.70(0.000)	0.67(0.000)	-0.42 (0.002)	-0.06 (0.505)	0.68(0.000)	0.58 (0.000)
Waist circumference	0.31 (0.02)	0.50(0.000)	-0.33 (0.016)	-0.24 (0.007)	0.51(0.000)	0.30(0.032)
FPG	0.10 (0.45)	0.21(0.016)	0.04 (0.745)	-0.06 (0.505)	0.10(0.25)	0.11 (0.22)
Serum Insulin	0.07(0.61)	0.49(0.000)	-0.02 (0.865)	-0.24 (0.007)	0.53(0.000)	-0.01 (0.90)
HOMA-IR	0.08 (0.572)	0.51(0.000)	-0.01 (0.946)	-0.21(0.016)	0.46(0.000)	0.01 (0.99)
TC	0.07(0.62)	0.34(0.000)	-0.11 (0.436)	-0.06 (0.486)	0.13(0.14)	0.09 (0.53)
TG	0.09 (0.51)	0.30(0.001)	-0.12(0.377)	-0.26(0.003)	0.20(0.02)	0.15 (0.27)
HDLc	0.09(0.53)	0.04(0.59)	-0.14 (0.325)	0.04(0.629)	0.10(0.265)	-0.08 (0.55)
LDLc	-0.02 (0.87)	0.31(0.000)	0.05 (0.707)	0.01(0.907)	0.14(0.11)	0.03 (0.81)
Omentin-1	-0.57(0.000)	-0.20(0.02)	1	1	0.14(0.12)	0.02 (0.85)
Adiponectin	0.46(0.001)	0.48(0.000)	0.02(0.85)	0.14(0.11)	1	1

The BMI-wise comparison displayed that serum levels of ADN and vaspin were substantially higher in the cases and control groups with BMI >25. No gender-wise difference was found in adipocytokines levels [not shown in the table].

Binary logistic regression analysis showed a significantly negative predictive relationship of vaspin and omentin-1 with DM (Table III).

**Table III:** Association of study variables with DM (Binary logistics regression analysis)*

*Variables*	*B*	*S.E.*	*Wald χ^2^*	*df*	*Sig.*	*Exp(B)*	*95% C.I. for Exp(B)*	

							*Lower*	*Upper*
BP Systolic	0.006	0.015	0.13	1	0.710	1.006	0.976	1.036
BP Diastolic	0.061	0.058	1.10	1	0.294	1.063	0.948	1.191
BMI	-0.046	0.106	0.185	1	0.667	0.955	0.776	1.176
Waist circumf	0.011	0.086	0.017	1	0.898	1.011	0.855	1.196
FPG	1.511	1.118	1.829	1	0.176	4.533	0.507	40.526
Serum Insulin	-0.129	0.385	0.112	1	0.738	0.879	0.414	1.868
HOMAIR	-0.485	1.552	0.09	1	0.755	0.616	0.029	12.897
TC	0.374	0.362	1.06	1	0.302	1.453	0.715	2.956
TG	2.411	.563	18.36	1	.000	11.144	3.700	33.568
HDLc	-1.652	1.503	1.20	1	0.272	0.192	0.010	3.643
LDLc	1.027	.446	5.30	1	0.021	2.792	1.165	6.690
Vaspin	-0.014	0.006	6.14	1	0.013	0.986	0.974	0.997
Omentin	-0.012	0.005	5.18	1	0.023	0.988	0.979	0.998
Adiponectin	0.437	0.389	1.26	1	0.262	1.547	0.722	3.316

* Parameters were analyzed to calculate the odds ratio in cases as compared to controls.

## DISCUSSION

Our study detected significantly lower serum omentin-1 concentrations, and it was inversely correlated with vaspin and no correlation with ADN among both groups. Lower serum omentin-1 was noted in the cases and control groups with >25 BMI, but this disparity was not noteworthy, and no gender-wise difference was found. Our study results are comparable with several investigations that described lower omentin-1 levels among T2DM.^11^,^12^ Literature indicates conflicting results such as a significant positive relationship between serum omentin-1 and BMI and body fat%^13^, a significant negative correlation with BMI, HbA1c, CRP, TC, LDLc, and TG, and notable positive relationship with HDL.^12^ A study described no significant link between omentin-1 and lipid profile, FPG, plasma insulin, and hs-CRP^14^, while another research observed its positive association with HbA1c and negative correlation with BMI, waist circumference, glucose, insulin, HOMA-IR, HDL, TG, DBP, and SBP.^15^

Similar to our results, two studies reported no change in levels of serum omentin-1 among lean and obese subjects in both groups^15^,^16^, and comparable to our results a meta-analysis stated a significant connection between DM and omentin-1.^12^ The declined concentration of serum omentin-1 in T2DM subjects indicates it is important for glucose metabolism. It has been reported that omentin-1 upsurges the transduction of insulin signals by triggering the protein kinase B and augments the entrance of insulin-facilitated glucose into adipocytes.^17^ It is suggested in the literature that lowered serum omentin-1 concentration affects insulin-mediated glucose entry.^18^,^19^ Different sample sizes and patients with co-morbidities acting as a confounding factor might have been reasons for inconsistent reported results.

Notably, lower serum vaspin was observed in the DM subjects compared to the control group, and it was inversely correlated with omentin and positively correlated with ADN among both groups. Various metabolic phenotypes were related to serum vaspin levels in patients and control subjects. The higher serum vaspin levels were noted in the subjects with BMI >25 in both groups. In binary logistic regression analysis, serum vaspin was found to be associated with DM. Several studies found vaspin levels lower^20^,^21^ and higher 22,23 in diabetic patients than in the healthy group.

Feng et al. (2014) also described higher vaspin concentrations in obese subjects than in the control group.^24^ On the contrary, no relationship between the vaspin level and obesity was demonstrated.^15^ A study in mice reported improvement in insulin sensitivity and glucose tolerance and after injection of vaspin.^25^ This shows that vaspin involvement in glucose metabolism. Yan et al. (2014) have reported that reduced vaspin concentration is a causative element for diabetes among non-diabetes and the progression of T2DM among DM patients.^26^ The variable results reported the relationship between vaspin anthropometric and metabolic parameters such as negative^21^, positive^22^, and no significant association.^14^

Our results regarding gender are similar to Chang et al. (2010)^27^, while dissimilar to a study that observed higher vaspin levels in females compared to males.^28^ We do not have any reason for no gender-wise difference of serum vaspin level. Overall, our results support the idea of the involvement of serum vaspin in T2DM, and binary logistic regression analysis further reinforced its association with T2DM. The differences in the present study and other studies may be attributed to genetic factors, size of the research population, different dietary patterns and geographical locations, and the use of different kits.

No significant variation in serum ADN levels was noted in the DM subjects than in the control subjects. Serum ADN levels were higher in cases and control groups with BMI>25, and no gender-wise change was noticed in ADN levels. Serum ADN levels were significantly correlated with few metabolic phenotypes in patients and the control group. We didn’t find any remarkable change in serum ADN levels in the DM subjects compared to the healthy group. Simultaneously, researchers have reported lower 22, higher 29, and no change^30^ in serum ADN levels among T2DM patients compared to the healthy group. Similar to our results, no gender-wise difference was reported by a Pakistani study.^31^ Possible reasons for the difference in ADN levels could be the participants’ different genetic makeup and illness levels.

### Limitations of the study

The smaller sample size is one of the limitations of this study. Moreover, being a cross-sectional study, no cause-and-effect conclusion be drawn.

## CONCLUSION

The DM group displayed substantially lower serum vaspin and omentin-1 levels. However, there was no consistent relationship observed between these adipocytokines and metabolic phenotypes. Further multi-centered studies on a larger scale are needed to identify these adipocytokines’ conclusive roles in T2DM.

### Authors’ Contribution:

**MB** Conceived the idea, did practical work, drafted manuscript and responsible and accountable for the work’s accuracy or integrity.

**ZJG, MAB, SHZ** Contributed to data collection and analysis, reviewed and edited manuscript.
